# Supplementary material for: Effectiveness of shared decision-making for glycaemic control among type 2 diabetes mellitus adult patients: A systematic review and meta-analysis
Source: PLoS One. 2024 Jul 31;19(7):e0306296. doi: 10.1371/journal.pone.0306296 (PMC11290692; doi:10.1371/journal.pone.0306296)
Supplement: S1 File — (DOCX) [file pone.0306296.s001.docx]

**Supplementary 1:** Electronic search strategy

**PubMed**: (((((((((((((((Effectiveness) OR (effects)) OR (impacts)) OR (influences)) AND (shared decision making)) OR (patient centered)) OR (patient involvement)) OR (patient engagement)) OR (patient empowerment)) AND (glycated hemoglobin)) OR (glycemic control)) OR (HbA1c level)) AND (diabetes)) OR (diabetes mellitus)) OR (Type 2 diabetes)) OR (T2DM)

**Search**: (((#1) AND (#2)) AND (#3)) AND (#4) Filters: Abstract, Full text, Clinical Trial, Randomized Controlled Trial, Humans, English Sort by: Most Recent

(("effect"[All Fields] OR "effecting"[All Fields] OR "effective"[All Fields] OR "effectively"[All Fields] OR "effectiveness"[All Fields] OR "effectivenesses"[All Fields] OR "effectives"[All Fields] OR "effectivities"[All Fields] OR "effectivity"[All Fields] OR "effects"[All Fields] OR ("effect"[All Fields] OR "effecting"[All Fields] OR "effective"[All Fields] OR "effectively"[All Fields] OR "effectiveness"[All Fields] OR "effectivenesses"[All Fields] OR "effectives"[All Fields] OR "effectivities"[All Fields] OR "effectivity"[All Fields] OR "effects"[All Fields]) OR ("impact"[All Fields] OR "impactful"[All Fields] OR "impacting"[All Fields] OR "impacts"[All Fields] OR "tooth, impacted"[MeSH Terms] OR ("tooth"[All Fields] AND "impacted"[All Fields]) OR "impacted tooth"[All Fields] OR "impacted"[All Fields]) OR ("influence"[All Fields] OR "influenced"[All Fields] OR "influences"[All Fields] OR "influencing"[All Fields])) AND ("hasabstract"[All Fields] AND ("clinical trial"[Publication Type] OR "randomized controlled trial"[Publication Type]) AND "loattrfull text"[Filter] AND "humans"[MeSH Terms] AND "english"[Language]) AND (("decision making, shared"[MeSH Terms] OR ("decision"[All Fields] AND "making"[All Fields] AND "shared"[All Fields]) OR "shared decision making"[All Fields] OR ("shared"[All Fields] AND "decision"[All Fields] AND "making"[All Fields]) OR ("patient participation"[MeSH Terms] OR ("patient"[All Fields] AND "participation"[All Fields]) OR "patient participation"[All Fields] OR ("patient"[All Fields] AND "engagement"[All Fields]) OR "patient engagement"[All Fields]) OR (("patient s"[All Fields] OR "patients"[MeSH Terms] OR "patients"[All Fields] OR "patient"[All Fields] OR "patients s"[All Fields]) AND "centered"[All Fields]) OR ("patient participation"[MeSH Terms] OR ("patient"[All Fields] AND "participation"[All Fields]) OR "patient participation"[All Fields] OR ("patient"[All Fields] AND "involvement"[All Fields]) OR "patient involvement"[All Fields]) OR ("patient participation"[MeSH Terms] OR ("patient"[All Fields] AND "participation"[All Fields]) OR "patient participation"[All Fields] OR ("patient"[All Fields] AND "empowerment"[All Fields]) OR "patient empowerment"[All Fields])) AND ("hasabstract"[All Fields] AND ("clinical trial"[Publication Type] OR "randomized controlled trial"[Publication Type]) AND "loattrfull text"[Filter] AND "humans"[MeSH Terms] AND "english"[Language])) AND (("glycated hemoglobin"[MeSH Terms] OR ("glycated"[All Fields] AND "hemoglobin"[All Fields]) OR "glycated hemoglobin"[All Fields] OR ("glycemic control"[MeSH Terms] OR ("glycemic"[All Fields] AND "control"[All Fields]) OR "glycemic control"[All Fields]) OR (("glycated hemoglobin"[MeSH Terms] OR ("glycated"[All Fields] AND "hemoglobin"[All Fields]) OR "glycated hemoglobin"[All Fields] OR "hba1c"[All Fields] OR "hba1cs"[All Fields]) AND ("level"[All Fields] OR "levels"[All Fields]))) AND ("hasabstract"[All Fields] AND ("clinical trial"[Publication Type] OR "randomized controlled trial"[Publication Type]) AND "loattrfull text"[Filter] AND "humans"[MeSH Terms] AND "english"[Language])) AND (("diabete"[All Fields] OR "diabetes mellitus"[MeSH Terms] OR ("diabetes"[All Fields] AND "mellitus"[All Fields]) OR "diabetes mellitus"[All Fields] OR "diabetes"[All Fields] OR "diabetes insipidus"[MeSH Terms] OR ("diabetes"[All Fields] AND "insipidus"[All Fields]) OR "diabetes insipidus"[All Fields] OR "diabetic"[All Fields] OR "diabetics"[All Fields] OR "diabets"[All Fields] OR ("diabetes mellitus"[MeSH Terms] OR ("diabetes"[All Fields] AND "mellitus"[All Fields]) OR "diabetes mellitus"[All Fields]) OR ("diabetes mellitus, type 2"[MeSH Terms] OR "type 2 diabetes mellitus"[All Fields] OR "type 2 diabetes"[All Fields]) OR "T2DM"[All Fields]) AND ("hasabstract"[All Fields] AND ("clinical trial"[Publication Type] OR "randomized controlled trial"[Publication Type]) AND "loattrfull text"[Filter] AND "humans"[MeSH Terms] AND "english"[Language]))) AND ((fha[Filter]) AND (clinicaltrial[Filter] OR randomizedcontrolledtrial[Filter]) AND (fft[Filter]) AND (humans[Filter]) AND (english[Filter]))
