# Supplementary material for: Effectiveness of shared decision-making for glycaemic control among type 2 diabetes mellitus adult patients: A systematic review and meta-analysis
Source: PLoS One. 2024 Jul 31;19(7):e0306296. doi: 10.1371/journal.pone.0306296 (PMC11290692; doi:10.1371/journal.pone.0306296)
Supplement: Attachment — Submitted filename: Response to Reviewers.docx [file pone.0306296.s003.docx]

**Responses to reviewers’ comments**

**Manuscript Title**: Effectiveness of shared decision-making for glycaemic control among type 2 diabetes mellitus adult patients: A Systematic review and Meta-analysis

Dear Editor,

Thank you for your suggested revision that has helped us to improve the manuscript. We have undertaken revision and modified the manuscript. We attached, you will find our revised and modified manuscript and we explained the substance modification and below you will find responses to each comment.

**Reviewer 1 Evaluation**

Dear reviewer 1, thank you for understanding our research work, your comments and suggestions. We attached, you will find our revised and modified manuscript and we explained the substance modification and below you will find responses to each comment.
Introduction:

1. Line 62,63 - Please be consistent in use of abbreviations –HbA1c or HbA1C

**Response** 1: Thank you, we have corrected it to HbA1c.

2. Line 64 -The statement given here seems irrelevant and the reference doesn’t match.

**Response 2**: Thank you, we checked it, and the irrelevant statement has been removed.

3. Line 66,67 - error in the statement. It should be HbA1c ≤ 7%.

**Response 3**: Now it has been Corrected.

4. Line 76 – the word "current" may be removed as the ADA guideline cited here was 2015 guidelines.

**Response 4**: Thank you, we have corrected.

5. Line 96 – repetitive word”in”

**Response 5**: we checked and corrected it.

6. Overall, the statements under introduction seem repetitive and the flow of language is not very professional and uncomfortable, and I would suggest making it more concise.

**Response 6**: Thank you. We have made it more concise through revision.

**Methodology:**

7. This systematic review is observed to have followed rigorous methodology, have applied relevant search strategies and analysis methods. The authors have provided an approved protocol (PROSPERO) along with the methods employed for article inclusion. The manuscript incorporates detailed descriptions of the search methodology, study selection, and data extraction procedures. PRISMA checklist, ROB for quality of included studies, Heterogeneity tests has been performed and shared.

**Response 7**: Thank you for your understanding of our research work.

**Methods:**

8. The types of studies included in this systematic review are thoroughly described. The PRISMA flow diagram is easy to follow and complete. ROB for the quality of studies was performed and the details are shared.
9. Line 177 – typo error

**Response 9**: Thank you, we corrected it.
Discussion and Conclusion:

10. Though the strength and limitations of the study are well thought and documented, the conclusion driven out of this study ( line 349-351) did not match the discussion points (line 330-332). I suggest to rewrite the discussion in a more intelligent way and draw precise conclusions.

**Response 11**: Thank you. We revised the discussion and conclusion parts.

**Reviewer 2 Evaluation**

Dear reviewer 1, thank you for understanding our research work, your comments and suggestions. We attached, you will find our revised and modified manuscript and we explained the substance modification and below you will find responses to each comment.

- The manuscript addresses a significant subject due to the progressive growth of the population with diabetes and its complications. It also emphasizes the importance of evaluating the shared decision-making for glycaemic control effectiveness, as it is widely recommended but not primarily followed in most places, and identifying what may influence its outcomes. However, this manuscript has some points that need to be clarified.

1. on page 3, line 66, it says ...'(HbA1c) ≥7% (53 mmol/mol) is considered a favorable indicator of glycemic control and is the recommended treatment a goal...'

**Response 1:** Thank you; it was an error, and now it has been corrected to HbA1c ≤7% (53 mmol/mol).

2. On page 5, lines 111-113, it says ...'the current systematic review and meta-analysis aimed to evaluate the effectiveness of shared decision-making compared to usual diabetes care for glycaemic control among type 2 diabetes mellitus adult patients.' Nevertheless, in the item 'Study selections and eligibility' on page 6, when you describe the 'C' of PICO criteria, it is not expressed that you will compare a group under usual care and another with SDM.

**Response 2**: Thank you, patients who received usual care of T2DM were comparison group (C ) and we have it made clear.

3. Related to observation #2, I wonder why the authors included in the review the study of Corser et al. (reference 26), which has only an intervention group evaluation and no control group with usual care. Moreover, this may interfere with the whole evaluation.

**Response 3**: Thank you. We excluded the study and performed a reanalysis; changes were made to the revised manuscript.

4. On page 9, Table 1, it says 18 RCTs, when it is 17; in the item Study events rate, there are no percentage results, and in the risk with usual care, the authors present the mean effect of SDM on HbA1C.

**Response 4**: Thank you. In our revised manuscript, we included 17 RCT studies, labeled Table 1 as Table 2, and moved the table to the end of the result section. The percentage results have been mentioned in the section on “participant characteristics,” and regarding the risk with usual care, we estimated the mean of HbA1c%, which was the primary outcome of the study, and the mean effect of SDM on HbA1c was estimated. The table has been updated and named Table 2, including details and clear information. To do so, we used a COCHRANE GRADE review to evaluate the certainty of the meta-analysis evidence from RCT studies.

5. The whole manuscript deserves a writing review.

**Response 5**: Thank you; we have revised the manuscript.
