# Supplementary material for: Effectiveness of shared decision-making for glycaemic control among type 2 diabetes mellitus adult patients: A systematic review and meta-analysis
Source: PLoS One. 2024 Jul 31;19(7):e0306296. doi: 10.1371/journal.pone.0306296 (PMC11290692; doi:10.1371/journal.pone.0306296)
Supplement: Attachment — Submitted filename: Response to Reviewers.docx [file pone.0306296.s004.docx]

**Manuscript title:** Effectiveness of shared decision-making for glycaemic control among type 2 diabetes mellitus adult patients: A Systematic review and Meta-analysis

**Manuscript number:** PONE-D-24-07517R1

**Response to Editor comments**
Journal Requirements: Please review your reference list to ensure that it is complete and correct. If you have cited papers that have been retracted, please include the rationale for doing so in the manuscript text or remove these references and replace them with relevant current references. Any changes to the reference list should be mentioned in the rebuttal letter that accompanies your revised manuscript. If you need to cite a retracted article, indicate the article’s retracted status in the References list and also include a citation and full reference for the retraction notice.

**Response**: Dear editor, thank you for the comment and suggestion to revise our manuscript to be considered for publication. Since we did not include and cite the retracted paper in our current review, no changes have been made to the cited references. To be sure, we have reviewed the references and rechecked for retraction status. We found a correction to reference number 17, and the correction to this article was published on February 14, 2024. The update did not affect the results of our current systematic review and meta-analysis. In addition, we have included the DOI for references cited when available.

**Response to reviewers’ comments**

**Reviewer** 1

Thank you for inviting me to re-review the manuscript “Systematic review and Meta analysis of literature on Shared decision-making for glycaemic control among type 2 diabetes mellitus adult patients”. The authors have taken the suggestions and comments from the previous review and have included. The introduction is more precise and to the point. Changes suggested to the methodology had been included and the results are discussed accordingly. PRISMA checklist on effectiveness of SDM included and very elaborate. The limitations of the study are well thought of and listed.
There are still more typo errors which were overlooked. I would suggest to meticulously go through the manuscript and correct the error.

**Dear Reviewer1:** Thank you for reviewing our manuscript and providing comments to improve and revise it. We checked all comments and suggestions; changes were made to the revised manuscript.

Some are:

1. Line 39 – reduction

**Response 1**: Checked and corrected.

2. Line 62 – glycated

**Response 2**: Corrected.

3. Line 80 – ‘between’ instead of ‘among

**Response 3**: Corrected.

4. Line 262,264 – HbA1c

**Response** 4: Correction made.

5. Line 289 – reduction

**Response 5**: Correction made.

6. Table 2 title – patients

**Response 6**: Corrected.

7. Line 337 – review, participated

**Response 7**: Correction made.

**Reviewer 2**
The authors answered all the reviewer's questions, and the included data helped better understand the study. However, there are still a few points to correct.

**Dear Reviewer 2**: Thank you for reviewing our manuscript and providing comments to improve and revise it. We checked all comments and suggestions; changes were made to the revised manuscript.

1. Fig 1 - PRISMA states 425 records, but on page 2, line 28, page 7, line 145, and page 9, line 193, it is mentioned as 445 records.

**Response 1**: Corrections were made to 425 records.

2. On page 17, line 337, two words need to be corrected.

**Response 2**: We checked them, and corrections were made.
