# Supplementary material for: Effectiveness of shared decision-making for glycaemic control among type 2 diabetes mellitus adult patients: A systematic review and meta-analysis
Source: PLoS One. 2024 Jul 31;19(7):e0306296. doi: 10.1371/journal.pone.0306296 (PMC11290692; doi:10.1371/journal.pone.0306296)
Supplement: Attachment — Submitted filename: Response to Reviewers.docx [file pone.0306296.s005.docx]

**Response to reviewers’ comments**

**Manuscript title**: Effectiveness of shared decision-making for glycaemic control among type 2 diabetes mellitus adult patients: A Systematic review and Meta-analysis

**Manuscript number**: PONE-D-24-07517R2

**Response to Editor comments**

**Journal Requirements**: Please review your reference list to ensure that it is complete and correct. If you have cited papers that have been retracted, please include the rationale for doing so in the manuscript text or remove these references and replace them with relevant current references. Any changes to the reference list should be mentioned in the rebuttal letter that accompanies your revised manuscript. If you need to cite a retracted article, indicate the article’s retracted status in the References list and also include a citation and full reference for the retraction notice.

**Response**: Dear editor, thank you for the comment and suggestion to revise our manuscript to be considered for publication. Since we did not include and cite the retracted paper in our current review, no changes have been made to the cited references in the revised manuscript.

**Response to reviewers’ comments**

**Reviewer 1:**

Reviewer #1: The authors have considered all the previous comments and has made changes accordingly except that there are still minor typographical errors which were overlooked:

Dear reviewer1: Thank you for reviewing our revised manuscript and providing comments to improve and revise it to be considered for publication. We checked all comments and suggestions; changes were made to the revised manuscript.

1. Line 123 – and google scholar

**Response 1**: Thank you, checked and corrected

2. Line 186 – analysis

**Response 2** : A correction has been made.

3. Table 2 – typo patients

**Response 3**: A correction has been made.

4. Line 339 - Typo review

**Response 4**: A correction has been made.

5. 344-346 – rewrite- "fair control"

**Response 5**: Thank you, changes were made.

**Reviewer 2:**

Reviewer #2: Dear Authors,
I am glad to re-review the manuscript "Effectiveness of shared decision-making for glycaemic control among type 2 diabetes mellitus adult patients: A Systematic Review and Meta-analysis," which addresses a subject that may contribute to improving diabetes outcomes. The authors accepted the reviewers' suggestions and have made the corrections.

**Dear reviewer 2**: Thank you for reviewing our revised manuscript and considering it for publication.
